# The entropic basis of collective behaviour

**DOI:** 10.1098/rsif.2015.0037

**Published:** 2015-05-06

**Authors:** Richard P. Mann, Roman Garnett

**Affiliations:** 1 Professorship of Computational Social Science, ETH Zurich, Zurich, Switzerland; 2 Department of Mathematics, Uppsala University, Uppsala, Sweden; 3 Department of Computer Science and Engineering, Washington University in St Louis, St Louis, MO, USA

**Keywords:** collective behaviour, maximum entropy, causal entropic principle, Galton–Watson process, Weber's law, entropic forces

## Abstract

We identify a unique viewpoint on the collective behaviour of intelligent agents. We first develop a highly general abstract model for the possible future lives these agents may encounter as a result of their decisions. In the context of these possibilities, we show that the *causal entropic principle*, whereby agents follow behavioural rules that maximize their entropy over all paths through the future, predicts many of the observed features of social interactions among both human and animal groups. Our results indicate that agents are often able to maximize their future path entropy by remaining cohesive as a group and that this cohesion leads to collectively intelligent outcomes that depend strongly on the distribution of the number of possible future paths. We derive social interaction rules that are consistent with maximum entropy group behaviour for both discrete and continuous decision spaces. Our analysis further predicts that social interactions are likely to be fundamentally based on Weber's law of response to proportional stimuli, supporting many studies that find a neurological basis for this stimulus–response mechanism and providing a novel basis for the common assumption of linearly additive ‘social forces’ in simulation studies of collective behaviour.

## 1. Introduction

Collective decision-making and the emergence of collective intelligence are key areas of study in animal behaviour and social science. Since Francis Galton observed the power of the central limit theorem to provide an accurate estimate for the weight of a bull by averaging individual opinions (as told by James Surowiecki [1]), the ability of groups to make decisions that improve on the accuracy of the individuals comprising them has continued to surprise researchers. Human [2], animal [3] and even algorithmic [4] groups have been shown to improve on individual performance in estimation problems (Galton's bull example), navigation [5], identifying superior options [6] and prediction tasks [7]. In an age of unprecedented global connectivity of individuals through Web and mobile Internet technologies, the opportunity to understand the origins of social behaviour is greater than ever before.

Much is already known about how the transfer of information by individuals can lead to intelligent outcomes on the group level. Models of social contagion [8–10], quorum decision-making [11–13], Bayesian social decision rules [14,15] and information cascades [16,17] all provide a detailed theory for how each agent in a group can acquire and use information from other individuals' actions, and under what conditions this leads to improved or disrupted decision-making.

However, when we face the challenge of understanding the collective behaviour of the millions of connected individuals now on our planet, the prospect of beginning that process at the level of a single individual decision maker is daunting. Statistical mechanics, and particularly the principle of maximum entropy [18], provides an expedient methodology for studying the behaviour of large systems with many interacting elements. Harte and co-workers [19,20] show how maximum entropy methods imply specific non-trivial distributions of organisms in space and energy usage, which match observed natural distributions and those predicted by more structured biological theory [21,22]. Maximum entropy distributions can also be used to analyse snapshots of moving groups, inferring effective interactions by assuming the snapshot of positions and directions is sampled from a Boltzmann distribution [23]. Such an approach assumes that the collective is in a form of quasi-equilibrium. Extending the maximum entropy concept, the principle of maximum entropy production (reviewed by Niven [24]) has enabled these methods to be applied to more-general flow systems outside of the classical notion of equilibrium, and causal entropy [25] has been proposed to extend this to the case where the individual elements of the system exhibit intelligence.

A physical, mechanical approach has already provided a fruitful route to understanding collective behaviour, in particular collective motion, via the abstraction of *social forces*: pseudo-forces that can modify an agent's energy depending on its alignment with or proximity to other individuals [26], or explicitly provide a physical force to alter the agents' motion [27,28]. Such approaches have been able to demonstrate why human and animal groups undergo phase transitions between quasi-equilibria analogous to those seen in statistical–mechanical systems and have been developed to a particularly high degree of sophistication in the study of human crowds [29], where they are used to understand disasters such as at Hillsborough (1989) and the Love Parade (2010) [30]. However, social forces are a convenient abstraction of psychological choices, and therefore are typically adjusted to fit observations, rather than being based on the fundamental logic of interactions.

In this paper, we demonstrate a new way to understand collective behaviour, from a purely entropic viewpoint, without any *a priori* specification of social information transfer, social forces or individual interaction rules. We do this by building on the causal entropic (CE) framework of Wissner-Gross & Freer [25]. By specifying our uncertainty about the long-term futures of a group of agents, we will show that the decisions this group makes *now* can be predicted. We will further show that social rules of interaction and social forces, as assumed in many studies of collective behaviour in the form of conditional expectations for agents to make specific choices based on their decisions of others, emerge not from the adaptiveness of the agents' choices, nor from any consideration of their immediate needs or desires, but simply from a tacit assumption that their long-term actions are maximally uncertain.

## 2. The causal entropic principle

The CE principle is an assertion about our knowledge of a system's future path through state space. This is fundamentally an argument from a principle of maximum *ignorance*—we deem ourselves to be as uncertain as possible about the path an agent will take through all the future options available. As we shall show, this counterintuitively provides us with information about which choices the agent is likely to make now. In previous work, Wissner-Gross & Freer [25] derived a ‘CE force’ that drives systems towards locally available new microstates that permit a greater number of available paths through future state space. In the cases presented by Wissner-Gross & Freer [25], this force acts upon particles moving in a continuous, bounded Euclidean space. As the ergodic principle for equilibria states that any microstate of the particles in the gas is equally probable, so in a causal entropic system, all available future paths are assigned an equal probability. Therefore, the probability of any new reachable microstate being selected is proportional to the number of future state-space paths that it makes available. This CE force was shown to cause a diverse range of systems to behave in apparently intelligent ways, mimicking for example animal use of tools or complex cooperation. Inspired by these examples, we consider whether the same principle can predict the interactions between individuals in groups that are the foundation of collective cognition and intelligence.

### 2.1. Application to collective decisions: a toy example

Consider observing a group of agents who must decide between two options, A and B. Typically for social animals (including humans) that live as groups, social interactions between individuals will have an influence on which option each agent chooses. This interaction is typically expressed via the conditional probability for a focal agent to choose option A, based on the number of other individuals who have chosen either A or B, which we denote *n*_A_ and *n*_B_: *P*(A|*n*_A_, *n*_B_). From this conditional probability, the likelihood *P*(*n*_A_) that a certain number of the agents will ultimately choose option A can be derived by considering the probability of all possible sequences of choices that lead to that outcome.

We address this problem in reverse. We first derive the group-level distribution *P*(*n*_A_), and ultimately use this to infer an equivalent individual interaction rule *P*(A|*n*_A_, *n*_B_) that satisfies this. We assume that at the macro-scale group level, the distribution of future paths through state-space will conform to the CE principle, and subsequently ask which interactions between individuals would need to evolve to produce this maximum entropy distribution. Thus, we retain the principle that the *individual* acts as the decision maker. Furthermore, we do not assign entropy-maximizing agency or will to the individual agent or to the group; we ask instead what interactions the maximum entropy distribution implies and assess whether these correspond to interactions previously observed in nature and in experiments.

**Figure 1.**
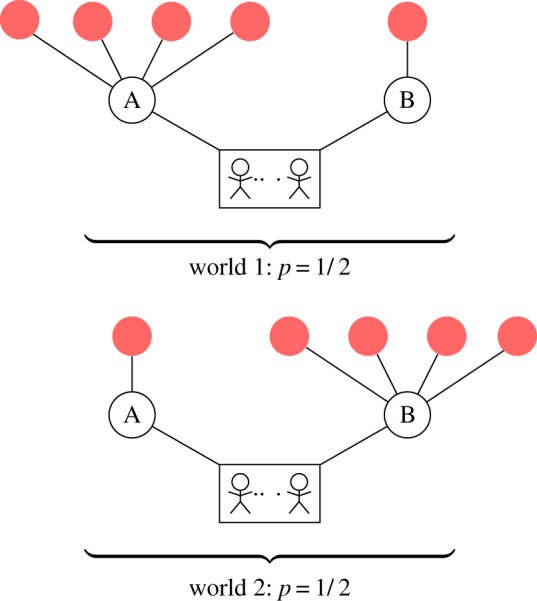
Schematic of a toy example illustrating the CE collective model. A group of agents at the root of the tree must choose between two options: ‘A’ and ‘B’. Two possible worlds exist: one where option A leads to four more choices and B to one, or one where A leads to one more choice and B to four. The decision rule for the group that maximizes their future path entropy averaged over the two possible worlds is a mixture of two binomial distributions, shown in figure 2.

In this example, we construct a hypothetical world where the information about the future is the following: behind one door lies four more options; behind the other, there is only one. This is illustrated in figure 1. Assuming that the door with four options is equally likely to be either A or B, what distribution of the agents between the two options will maximize their expected entropy, over the possible future paths to the final level of the branching tree?

For any given branching tree, entropy is maximized by making any assignment of the agents to each future path that reaches the final level equally probable. Because the graph of choices is a tree, each final option is associated with a single unique path through the future space; therefore, it is equivalent to assign agents randomly to the final nodes on the tree. We aim to find a consistent distribution of agents that maximizes the path entropy over all possible worlds—a general way for the agents to organize themselves such that their entropy will be as high as possible, on average, in all the worlds they might encounter. Therefore, we take each possible tree, weighted by its probability of existing and assign a uniform multinomial distribution of the agents to its final nodes. We then feed this distribution back to the first choices (in this case, A and B) that the agents must make. Denoting by *N* the total number of agents, and by *n*_A_ and *n*_B_ the number choosing each door, this model implies that the probability distribution for the number choosing door A is a weighted sum of two binomial distributions, one with *p* = 4/5, the other with *p* = 1/5. Each has a weight of 1/2, as each has a 50% chance of existing:2.1
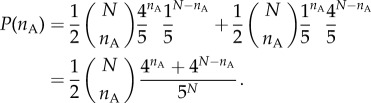


**Figure 2.**
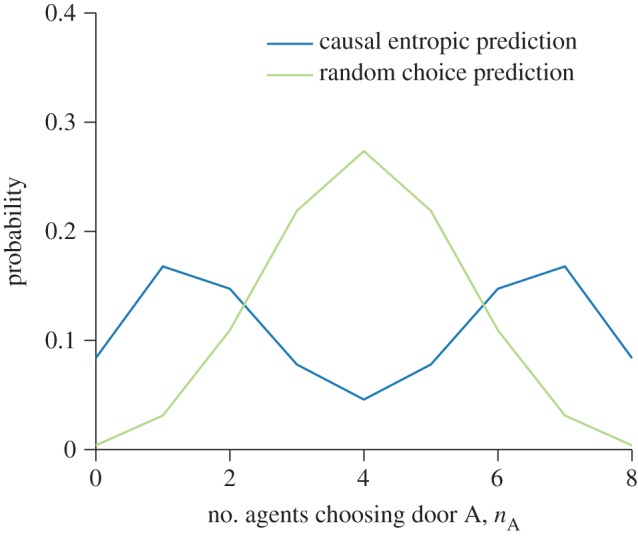
An example of predicted decisions by a group of eight agents in a ‘toy’ world: choosing one (unknown) option leads to four possible future paths, and the other to one. A future path is assigned at random to each agent, averaging over possible configurations of the future world, where the four options may be behind choice A or B. The predicted distribution of *n*_A_, the number of agents choosing door A, is a weighted sum of binomial distributions, with far greater cohesion than expected if each agent would independently choose a door at random.

For the case of eight agents picking between these two options, the expected distribution is shown in figure 2, alongside the distribution we would expect if each agent chose a door independently at random. The exact form of the distribution varies with the total number of agents, as well as with the number of future options. The figure clearly shows that the CE principle, picking randomly from future options rather than the immediately available ones, induces a greater degree of cohesion on the agents—they are much more likely to choose the same option. This cohesive ‘force’ increases as the difference between the number of options behind each door increases.

## 3. Collective causal entropic model

We now expand the toy example above to consider more general collective decisions, where the information about the number of future options is less precise. Letting *P*(*π*_A_) and *P*(*π*_B_) describe the probability of finding *π*_A_ and *π*_B_ future paths behind doors A and B, respectively (assuming for now that these are independent), equation (2.1) generalizes to an infinite sum of probability-weighted binomial distributions.3.1
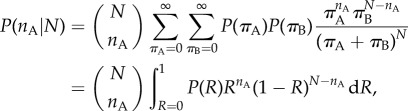
where *R* = (*π*_A_)/(*π*_A_ + *π*_B_). The key factor in equation (3.1) that controls the number of agents *n*_A_ choosing door A is the ratio *R*, the proportion of future options that lie behind door A. The problem of estimating the agents' behaviour is thus largely a problem of estimating *P*(*R*), the probability of this ratio.

### 3.1. A distribution for the number of possible futures

**Figure 3.**
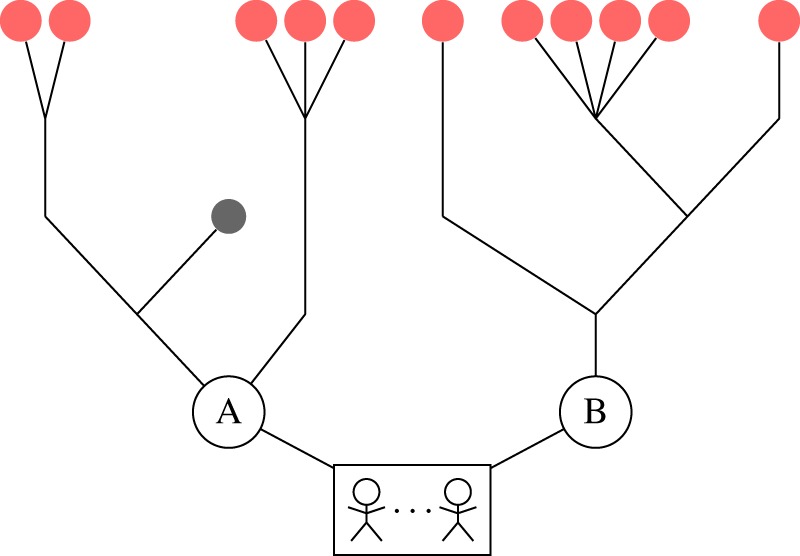
Schematic illustrating the general branching process of future choices. Each choice leads to an unknown number of future options to choose between, creating an expanding tree of possible future paths. The number of new options is generated from a stationary probability distribution, such that each new branch forms an independent and identically distributed tree. The total number of options at the top of the tree, *π*, is distributed according to a GW process. If the probability of generating no new choices is non-zero, dead-ends can form (black circle) and there is a probability *α* that the tree will become extinct. The CE collective model assumes that agents will be uniformly distributed on the final options (red circles), weighted by the probability of the tree being generated by a GW process.

In general, the number of future paths that either A or B may lead to may take any distribution. However, for the purposes of deriving the consequences of a model of collective decision-making, we must determine a specific form for *P*(*π*_A_) and *P*(*π*_B_), and most importantly for *P*(*R*). We propose the following method: a continuing branching tree of possible choices, in which each branch leads to an unknown number of future choices (illustrated in figure 3). The number of new choices generated on each branch is determined by some fixed distribution, independent of time. This is a Galton–Watson (GW) process [31]. We stress that the agents themselves need not hold any beliefs about these future choices. Instead, we argue that agents will develop interaction rules that serve to maximize entropy over these possible trees of future choices.

We are interested in the number of nodes on this branching tree after some time window *h*—the height of the tree. The Kesten–Stigum theorem [32] states that for any GW process, the distribution on the number of nodes converges to an exponential distribution, conditional on the tree not becoming extinct. Thus, including the possibility of extinction, for large *h*, the number of options is distributed as3.2
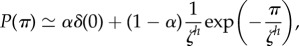
where *ζ* is the mean number of descendants of each node in each generation, *α* is the extinction probability and *δ* is the Dirac delta function. The extinction probability is determined by the fixed point of the generating function for the number of new choices generated on each branching. We will treat *α* as an adjustable parameter of our model. The behaviour of agents on this tree is determined, via equation (3.1), by the ratio distribution *P*(*R*) = *P*((*π*_A_)/(*π*_A_ + *π*_B_)). Since our assumption is that each new branch of the tree forms an independent GW process, this takes a simple form3.3
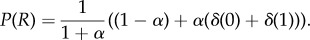
This follows from noting that the ratio *X*/(*X* + *Y*) of two identically distributed exponential random variables *X* and *Y* is a uniformly distributed random variable on (0, 1), and considering the special cases where either *π*_A_ or *π*_B_ is zero. Instances where both *π*_A_ and *π*_B_ are zero are undefined and do not contribute to the calculation. The Dirac delta functions are the result of the possible extinction of one branch or the other. The final distribution over the choices of *N* agents can be obtained via equation (3.1) and mirrors the distribution of *R*, with an equal probability of 1 to *N* – 1 agents choosing door A, and higher probabilities for either 0 or *N* agents to do so if *α* > 0. An equivalent model exists for a tree embedded in continuous time: the Yule process [33,34]. Thus the distribution derived for *P*(*R*) does not depend on whether the branching process for possible future trees is discrete or continuous in time.

### 3.2. More than two choices

The same principles used to derive the distribution of the agents over two choices can be applied to an arbitrarily higher number of options. To do so, we need the following fact: the proportional ratios of i.i.d. exponential random variables *X*_1_,*X*_2_, … ,*X_K_* are beta distributed. Using this fact and accounting for the probability that one or more of the trees behind each option goes extinct, we may generalize equation (3.3). We have the following probability distribution for *R*, the proportion of future paths behind one choice, in the case where there are *K* options:3.4
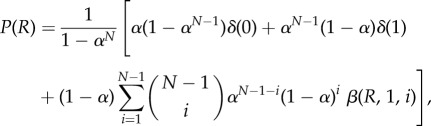
where *β*(*R*; *a*, *b*) represents the beta probability distribution on *R* with parameters *a* and *b*. As *α* becomes large, the factors multiplying the beta probability distributions tend to zero faster than the delta function terms, and consensus is still enforced. Each door shares an equal probability of being the consensus choice, reflected in the greater chance that *R* = 0 than *R* = 1. This result mirrors the experimentally observed tendency of, for example, fish to remain as a group when presented with three options [35], though it should be noted that this framework does not provide a clear way to model groups with conflicting preferences—a limitation addressed in the discussion. For all but the highest values of *α*, the probability of a consensus decreases with the number of options *K*, implying the common-sense notion that the probability of all agents choosing the same option is reduced as the number of equivalent choices becomes very high.

## 4. Consequences

### 4.1. Consensus decision-making

**Figure 4.**
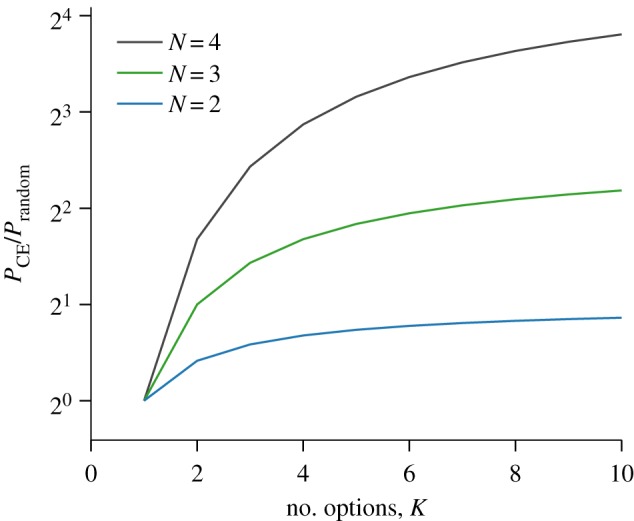
The ratio of the probability that a group of *N* agents make the same choice from *K* options within the CE model, relative to random chance. Note the log scale on the *y*-axis. The ratio is always above one for *K* > 1 and increases with both *K* and *N*, indicating the CE model's bias towards consensus collective decision-making.

The CE model predicts a tendency for groups of agents to reach a consensus. In the case where the extinction probability is greater than zero, there is a strong entropic ‘bonus’ for agents to remain as a single group, specified by the Dirac delta functions in equations (3.3) and (3.4). However, even in the case where the probability of all but one future tree becoming extinct is effectively zero, such as when *α* is zero or very small, or when the number of choices is very high, there is still a strong bias towards consensus decisions. For example, in the case that *α* = 0, in figure 4 we plot the probability that a group of agents of size 2, 3 or 4 will choose the same option from *K* independent choices, compared to the probability of this occurring if each agent makes a choice uniformly at random.

### 4.2. Social interaction rules

The entropic prediction of collective consensus is fundamentally a group-level analysis. Most studies in collective decision-making have started from a model of how individuals react to the decisions of others. What individual interaction rules would be necessary to produce the group-level behaviour that our analysis predicts? We can answer this question by considering a single individual choosing from two options when the other members of the group have already decided. From equation (3.1), assuming that *n*_A_ and *n*_B_ agents have already assigned themselves to options A and B:4.1
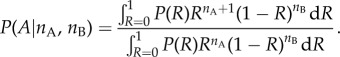


If the extinction probability is zero (*α* = 0), implying that *P*(*R*) = 1, this can be simplified in form to reveal a Weber's law interaction [36]:4.2
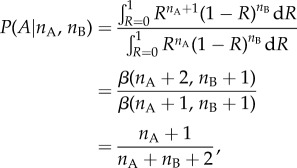
where *β* is the beta function. This is Weber's law with one ‘pseudo-observation’ for both A and B, and also corresponds to the expected value of a Bernoulli probability after observing *n*_A_ successes and *n*_B_ failures, assuming a uniform prior.

In the case where *α* > 0, there exists a special case when either *n*_A_ or *n*_B_ is equal to zero. For example, if *n*_A_ > 0 and *n*_B_ = 0 then4.3
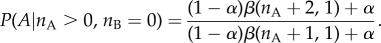


In this special case, *α* → 1 enforces the same consensus as derived at the group level, since the first agent to commit to option A or B makes the probability of that option for subsequent agents approximately equal to one, thus causing an irreversible information cascade.

### 4.3. Collective intelligence

The entropic enforcement of consensus decisions implies some degree of collective intelligence. To see this, consider the model used by Ward *et al.* [37] to explain the collective decisions of groups of varying size. In this ‘many-eyes’ model, if any one agent in a group spots a threat, all agents will avoid it. This implies that the proportion of agents avoiding a threat should grow in proportion to the probability that at least one will spot the threat, i.e. 1 − 0.5(1 − *d*)*^N^*, where *d* is the detection probability.

Our model implies a similar result. As the agents themselves are not *actively* trying to maximize entropy (instead, social decision rules have evolved that tend to maximize entropy in general), any agent seeing a threat should avoid it. However, once this occurs, the general tendency of the other agents to maintain a consensus means that the group will generally stick together, with a probability determined by the extinction probability of the branching process, closely mimicking the many-eyes model. We can calculate the expected number of agents avoiding a threat as a function of the extinction probability, by conditioning equation (3.1) on a given number, *i*, detecting the threat and avoiding it, and weighting by the probability of that number of detections, given *d*.4.4
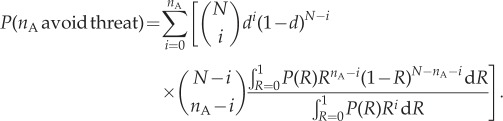


**Figure 5.**
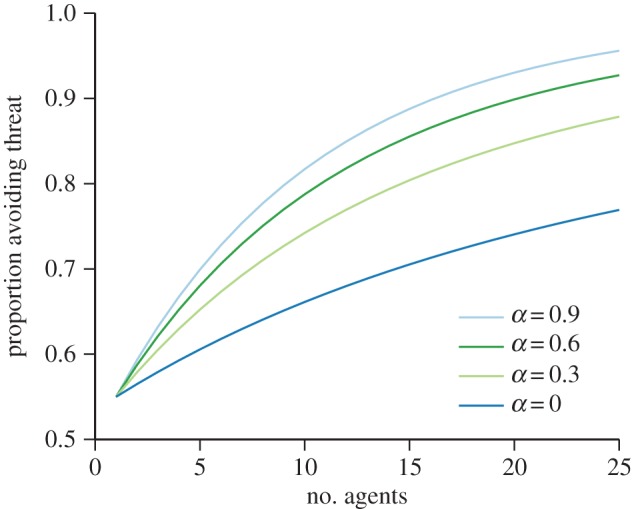
The increasing proportion of agents avoiding a threat with a low detection probability (*d* = 0.1) as a function of group size, for different values of the extinction probability on the future paths tree. The greater the possibility of one or other of the future path trees becoming extinct, the greater the cohesive force between the agents, and thus the stronger the information transfer between the detecting agents and the others, resulting in improved collective intelligence.

In figure 5, we plot the implied collective intelligence for different values of the extinction probability, in the case where any given agent has a *d* = 0.1 chance of detecting a hidden threat, as in the example of Ward *et al.* [37]. The prediction for high values of *α* is essentially identical to the prediction of Ward *et al.* [37]. When *α* is high, consensus is entirely enforced since equation (3.3) tends to the sum of two delta functions. This implies that one agent that spots and avoids the predator is sufficient to cause all group members to avoid it, matching the assumption made by Ward *et al.* [37].

### 4.4. Collective motion: derivation of a social force

Set in a discrete space, the model we describe here does not give immediate quantitative predictions about the types of collective motion [38] we would expect in a continuous space. However, we can sketch what such a generalized model would look like. The choice of which direction to move in is a decision like any other, but with many possible options. According to the arguments above, agents in approximately the same spatial location—those who will experience the same branching process of future options—should tend to move in the same direction.

For a given type of agent and environment, there is likely to be a typical spatial range over which future option trees are strongly correlated. We can associate this with the zones of interaction found in many models of collective motion, such as the classic Vicsek model [26] and Couzin zonal model [27]. In the case of a relatively confined environment, individuals outside of the immediate perceptual range may still experience the same future trees, and this can be expressed in individual interaction terms via a memory of encounters [39], leading to something akin to a mean-field model.

We can discuss the form of interaction rules with reference to a quantitative special case. Assume that in a continuous space, there are *N* + 1 agents, of which *N* are already committed to a particular position. Where should the (*N* + 1)th agent position itself? Let *R_i_* = *π_i_*/∑*_j_π_j_* be the ratio of number of future options at *x_i_* to the number available at all other points {*x_j_*}. At each point *x_i_* in the space-direction continuum occupied by an agent in {1, … ,*N*}, there is a probability distribution over this ratio *P*(*R_i_*|*x_i_*), where *R_i_* are assumed to be i.i.d. unless two points share the same future tree. Let us further assume a particular form for the distribution *P*(*R_N_*_+1_|*x_N_*_+1_): with probability *γ_k_*, this position shares a future tree with position *x_k_*. We take this probability to be defined by a squared exponential decay function4.5
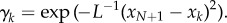


The probability distribution of possible position choices *n_N_*_+1_ is determined by a mixture of the possibility that *P*(*R_N_*_+1_|*x_N_*_+1_) is independent of all other points, and each of the possibilities that *x_N_*_+1_ shares a future tree with the position of another agent. Again ignoring second-order effects, we have4.6
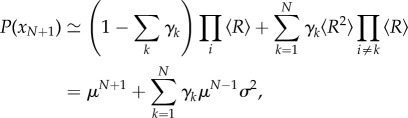
where 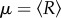 and 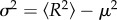. We find the optimal position by maximizing *P*(*x_N_*_+1_), obtaining4.7
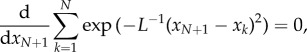
which we identify as the least-squares solution: 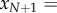 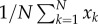. Therefore, the unique optimal position for agent *N*+1 is the mean position of all the other agents, implying a social force towards this point proportional to d*P*(*x_N_*_+1_)/d*x_N_*_+1_. It should be clear that the same argument would apply in relation to the direction choices of other individuals as well, creating an equivalent social force to rotate the agent's direction towards the average of the group. If the probability of sharing future trees were correlated between space and direction, then a distance-dependent alignment force would emerge.

## 5. Discussion

We have demonstrated that the CE principle gives a purely statistical prediction for many of the emergent properties of collective behaviour, without any detailed understanding on the mechanisms of interactions between individuals. Compared to previous work applying maximum entropy methods in behavioural ecology (e.g. [20,23]), our approach differs by focusing on dynamical processes rather than static snapshots or equilibrium distributions. As such we measured entropy over paths through state space, following Wissner-Gross & Freer [25], rather than entropy over current positions and velocities. Adopting the taxonomy of modelling approaches described by Sumpter *et al.* [40], this is a purely global approach to modelling groups and is complementary to a detailed understanding of individual behaviour, rather than a replacement. On the individual level, selection favours those who make decisions which aid their survival and reproduction. This is entirely consistent with the idea that the group operates with some degree of consensus, as following the decisions of other group members is often individually rational [14–16]. Our claim is that the resulting collective behaviour can be understood in part from a group-level entropic view without a detailed understanding of how or why individuals interact by considering the probability distribution of all possible futures for the group.

Our model takes a unique approach to understanding the origins of collective behaviour and makes testable predictions about the fundamental form of social interactions. It predicts that interactions between individuals take the form of Weber's law. This social decision rule has empirical support in the response to various stimuli of several species, e.g. insects [41,42], fish [15] and humans [10,43], as well as a solid grounding in experimental psychology [44] and the psychophysical [45] and neurological [46] basis of estimating differences. In continuous spaces, we have shown that reasonable assumptions about the spatial and directional correlations between individuals' futures lead to social forces resembling those of self-propelled particle models, which have also found experimental support [47–49], and which underlie static maximum entropy approaches to collective self-organization [23]. As our model can be shown to be equivalent to Weber's law interactions and social forces, data supporting these form of interaction would equally support our construction in an empirical test.

However, there are other studies that find that individual decisions are better described by more nonlinear interactions [11,12]. As pointed out by Bialek *et al.* [23], the fact that a maximum entropy method makes minimal assumptions does not necessarily make it correct. Instead, this should be seen as a basis model for social behaviour, implying that Weber's law can be considered the most basic form for social interactions. However, observations of apparent nonlinear interactions do not necessarily imply a fundamentally different mechanism. Perna *et al.* [42] have shown that an accumulation of Weber's law interactions, combined with some degree of noise or inaccuracy (which we would expect in any real system) can lead to apparently nonlinear interactions. We therefore suggest that where nonlinear interactions are observed, these may be the result of an accumulation of smaller scale linear interactions, which self-propelled particle models have shown can lead to strongly nonlinear consensus decision-making in moving groups [50,51]. In many experimental set-ups involving animal groups, the choices ultimately made by the individuals are not single events, but the final result of a period of motion where many smaller choices are made, supporting the idea that the final choice can be seen as an accumulation of smaller interactions.

A limitation of our model is the lack of a description of groups with conflicting information or preferences. Variation in information or personality in groups has been shown to be a potentially important driver of collective outcomes [50,52,53]. This could potentially be addressed by assigning different beliefs to each agent about the probability distribution on future trees. However, we have deliberately framed our model in terms of a consistent rule that produces a maximum entropy result over all possible futures, rather than assigning entropy-maximizing agency to the individuals themselves. There is no clear reason for individual agents, animal or human, to *desire* greater entropy over future paths; rather, we consider it as a minimal assumption regarding our certainty in which futures may be possible, and which decisions the agents will take. Nonetheless, the viewpoint could be relaxed to allow the emergence of a more sophisticated model including conflicting groups in the future. The entropic consequences of conflict are therefore an area of importance for future research in this area.

The model described here gives a simple caricature of the types of decisions that face groups of intelligent agents. This abstraction is useful for understanding the logic of how CE maximization implies group behaviours, social interactions and collective intelligence. We have shown how the model might be generalized to a continuous space in consideration of collective motion. Such an expansion of the model could potentially describe the structure of moving animal groups [23,54,55] and patterns of group-level direction changes [56]. More widely, the CE principle may provide a general framework for understanding the dynamics of complex intelligent systems, extending from animal groups, through organizations such as corporations and governments, to global human social systems built on the enormous connectivity of the Internet. We cannot be sure what series of choices every animal, pedestrian, bureaucrat or social-network user will face, or what decisions they will make, over an extended period of time. But precisely this ignorance can help us to predict what they will do next.
